# Preimplantation Genetic Testing Inhibits the Transmission of Pathogenic Variants Associated With Cerebral White Matter Disease

**DOI:** 10.7759/cureus.65164

**Published:** 2024-07-23

**Authors:** Xianjing Huang, Pingping Qiu, Hong Ji, Yingying Shi, Ling Zhang, Longmei Wang, Libin Mei, Ping Li

**Affiliations:** 1 Department of Reproductive Medicine, The Affiliated Women and Children's Hospital of Xiamen University, Xiamen, CHN; 2 Xiamen Key Laboratory of Reproduction and Genetics, The Affiliated Women and Children's Hospital of Xiamen University, Xiamen, CHN

**Keywords:** snp-array, next generation sequencing (ngs), preimplantation genetic testing, notch3 gene, abcd1 gene, cerebral white matter disease

## Abstract

Hereditary white matter disease is a series of progressive genetic diseases that mainly affect the white matter of the central nervous system. The development of molecular genetics enables the clinical diagnosis, carrier detection, and prenatal diagnosis of hereditary white matter disease. Here, we block the transmission of pathogenic variants in ABCD1 and NOTCH3 in a family with cerebral white matter disease via preimplantation genetic testing (PGT). Pathogenic genes were identified based on clinical manifestations, genetic background, and the results of targeted gene capture sequencing. A blastocyst biopsy was performed, and multiple annealing and looping-based amplification (MALBAC), next-generation sequencing (NGS), and single nucleotide polymorphism (SNP) arrays were used to analyze ploidy and the state of the gene mutations. The proband (III:1) had hemizygous mutations in ABCD1 (c.323C>A (p.Ser108 *) and c.775C>T (p.Arg259Trp)) and heterozygous mutations in NOTCH3 (c.1630C>T (p.Arg544Cys)), which were maternally inherited (II:2). After genetic analysis, a euploid blastocyst without ABCD1 and NOTCH3 variations was transferred. A healthy male baby was born at full term, and the results of prenatal diagnosis by amniocentesis in the second trimester verified the results of PGT. To our knowledge, this is the first report of simultaneously blocking the transmission of pathogenic variants in ABCD1 and NOTCH3 via PGT. This report highlights the feasibility and effectiveness of PGT in preventing cerebral adrenoleukodystrophy (cALD) and cerebral autosomal dominant arteriopathy with subcortical infarcts and leukoencephalopathy (CADASIL) and provides valuable insights for the diagnosis and treatment of similar cases.

## Introduction

Cerebral white matter disease is a demyelinating disease of the central nervous system that is caused by various factors, including genetics, infection, poisoning, and vascular diseases. It usually consists of two categories: primary and secondary. Based on the maturity of the myelin sheath, primary white matter diseases are divided into congenital and acquired diseases. Cerebral white matter disease primarily clinically manifests as mental state alterations such as deficiencies in attention, memory, visual-spatial skills, executive function, and emotional self-regulation.

Adrenal leukodystrophy (ALD), a progressive neurodegenerative disease with a high degree of clinical heterogeneity, is caused by defective peroxisomal β-oxidation of very long-chain fatty acids (VLFA). Adrenal leukodystrophy mainly affects the adrenal gland, spinal cord, and central nervous system. Based on the age of onset and clinical manifestations, ALD is classified into three clinical types: cerebral adrenoleukodystrophy (cALD; childhood, adolescent, and adult types), adrenomyeloneuropathy (AMN), and Addison’s disease [1]. Cerebral adrenoleukodystrophy, characterized by cerebral demyelination, is commonly found in the corpus callosum and leads to motor disorders, memory loss, and seizures. Cerebral adrenoleukodystrophy commonly occurs in childhood or adolescence. Adrenomyeloneuropathy, characterized by axonal lesions in the spinal cord and peripheral nerves, primarily presents as progressive weakness in both lower limbs, muscle spasms, and an abnormal gait. Most patients with AMN develop symptoms in adulthood, and some can develop into cALD. Addison's disease, characterized by adrenal cortical dysfunction, primarily manifests as darkening of the skin, halophilia, excessive sweating, fatigue, weakness, frequent vomiting, diarrhea, and syncope. The gene involved in ALD pathology, ABCD1, located at Xq28, encodes the adenosine triphosphate (ATP)-binding cassette subfamily D member 1 (ABCD1) protein. The ABCD1 protein is a peroxisomal transmembrane protein that transports VLCFA CoA from the cytoplasm to the peroxisome, utilizing the energy generated by ATP hydrolysis [2]. Functional defects in ABCD1 lead to the accumulation of VLCFA in cells, ultimately leading to ALD. Thus, plasma VLCFA serves as a diagnostic marker for ALD. Currently, most ALD treatments are effective only during the early disease stage; however, owing to rapid progression, no effective treatment exists for the treatment of severe cALD [1].

Cerebral autosomal dominant arteriopathy with subcortical infarcts and leukoencephalopathy (CADASIL) is the most common genetic cause of small vessel disease [3] which predominantly clinically manifests as recurrent ischemic stroke, transient ischemic attacks, migraine with aura, progressive white matter lesions, memory loss, cognitive decline progressing to dementia, mood disturbances, apathy, and multiple psychiatric symptoms [4,5]. The age of CADASIL onset varies from infancy to adulthood, though most cases occur during early adulthood [4]. CADASIL is caused by mutations on the NOTCH3 gene mapped to chromosome 19p13.1. The NOTCH3 gene encodes the NOTCH signaling receptor, which is mostly expressed in vascular smooth muscle cells and pericytes, performs essential developmental functions, and is involved in tissue maintenance and renewal [4,6]. To date, no therapeutic options for CADASIL are available, except for a few symptomatic treatments.

While preimplantation genetic testing for monogenic disorders (PGT-M) has the potential to prevent future progeny from being affected by genetic conditions, it is the best strategy to avoid the physical and psychological trauma of pregnancy termination. It has been performed worldwide since it was first used by Handyside et al. in the United Kingdom to sex embryos in 1990 [7]. With the development of molecular biology, the accuracy and safety of PGT have improved, and the selection strategy for embryos has been optimized. In this study, we provide accurate preimplantation genetic testing results for a family with cerebral white matter disease, successfully preventing vertical transmission of ABCD1 and NOTCH3 mutations within the family.

## Case presentation

A 35-year-old Chinese female (Ⅱ:2, Figure 1) delivered a male infant (Ⅲ:1) in 2014 who presented with understanding and memory decline, retreat from learned life skills, and a tendency to fall while walking at seven years old. A brain MRI of the proband revealed white matter lesions. Subsequent targeted gene capture sequencing identified two hemizygous variations in ABCD1(NM_000033.3):c.323C>A(p.Ser108*) and c.775C>T (p.Arg259Trp,rs201118034) (Figure 2A) and a heterozygous variation in NOTCH3(NM_000435.2): c.1630C>T(p.Arg544Cys) (Figure 2B) in the proband. The proband’s father (II:1) was healthy and did not present with any clinical alterations. To avoid the birth of another child with white matter disease, the couple sought PGT-M combined with assisted reproductive technology (ART) treatment at the Reproductive Medicine Department of Xiamen Women and Children’s Hospital in Xiamen, China.

**Figure 1:**
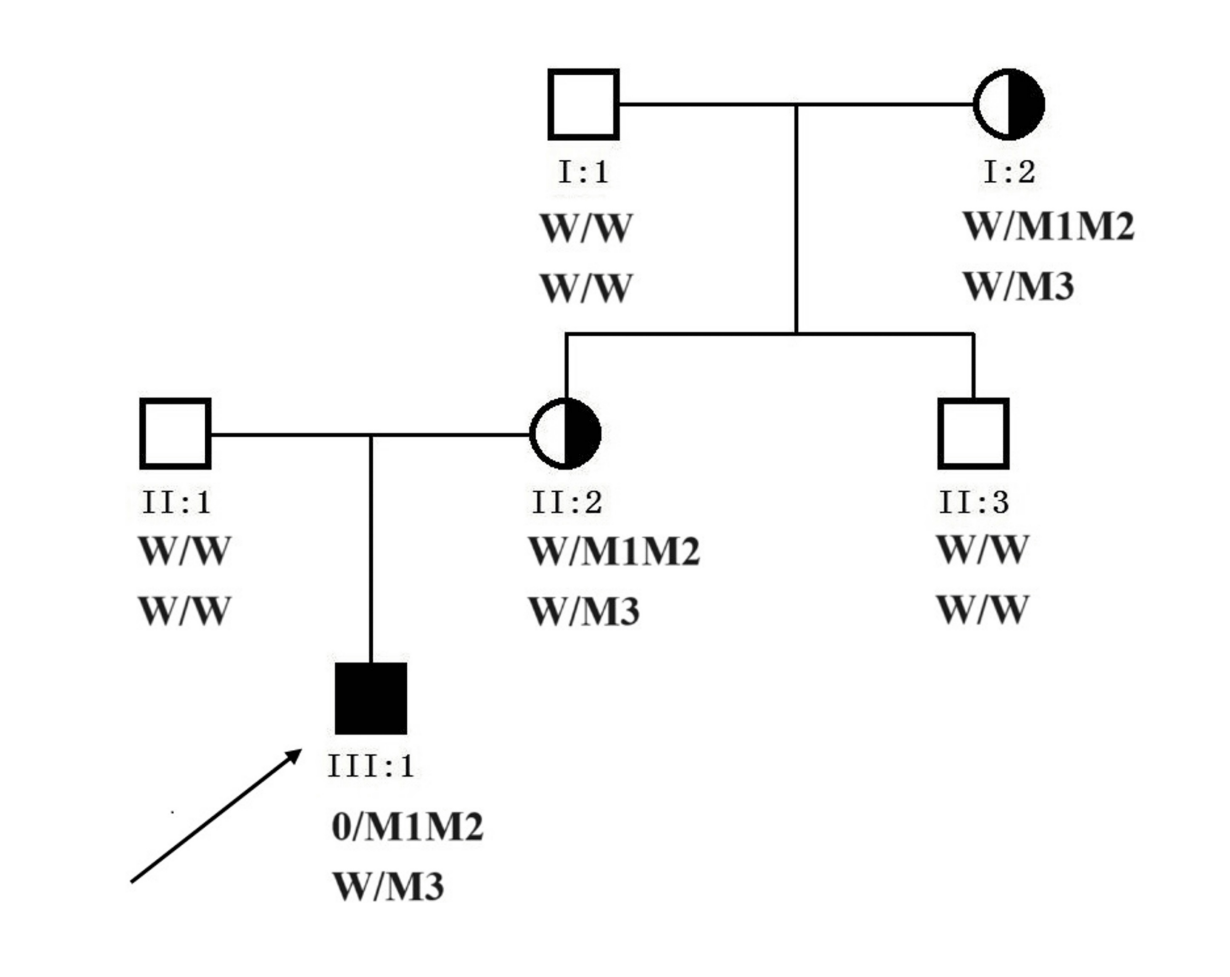
Pedigree of the family with cerebral white matter disease W: wild type; M1: ABCD1 c.323C>A mutation; M2: ABCD1 c.775C>T mutation; M3: NOTCH3 c.1630C>T mutation. 0/M1M2 means hemizygous variation. The arrow identifies the proband. The schematic diagram was drawn by Cyrillic, version 2.02 (AP Benson, London, UK).

**Figure 2:**
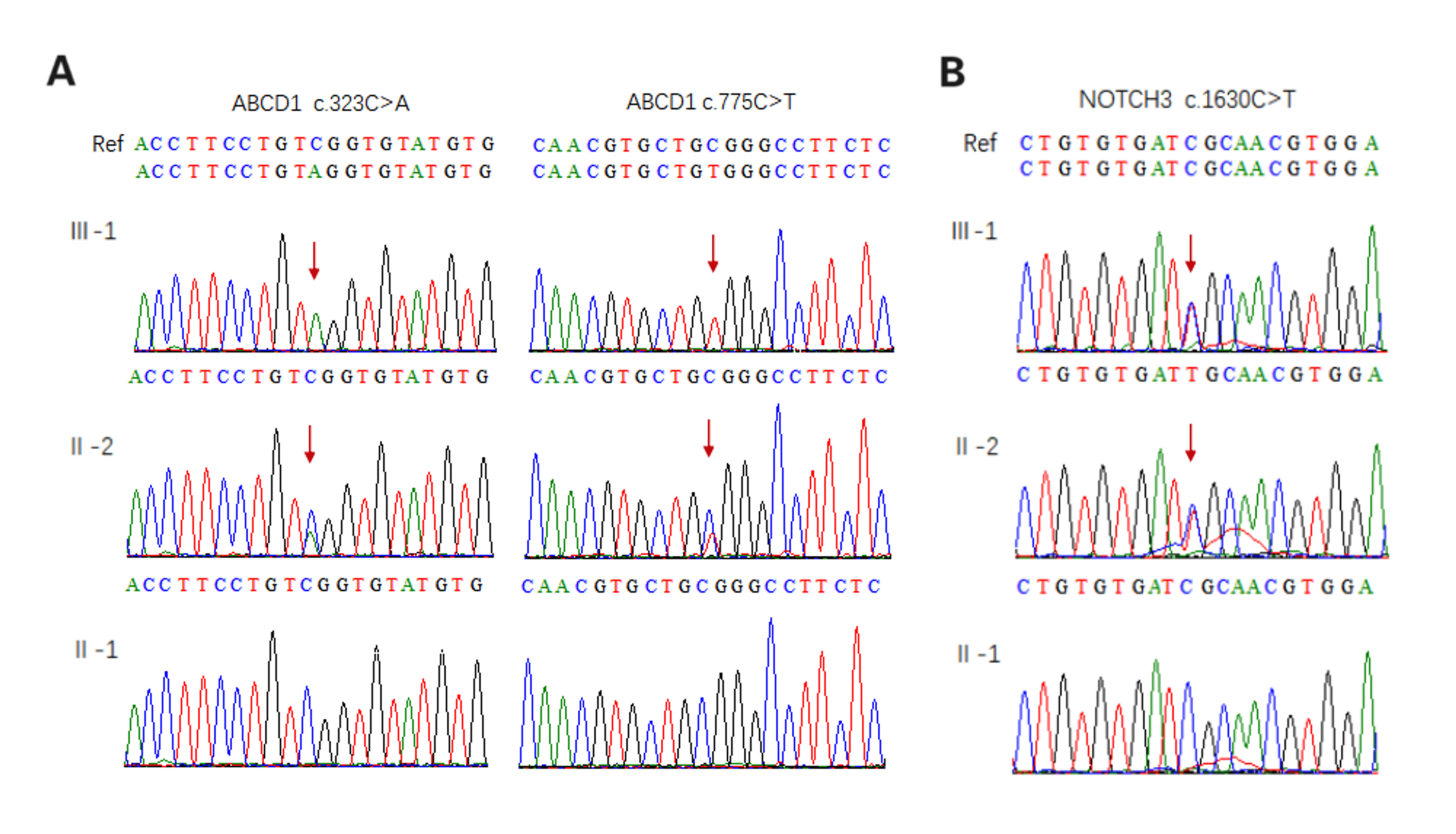
Validation of the mutation locus in the family A: identifying mutations in ABCD1; B: identifying mutations in NOTCH3. Sanger sequencing results show that the proband (Ⅲ:1) carried ABCD1 c.323C>A and c.775C>T and NOTCH3 c.1630C>T, which were inherited from the mother (Ⅱ:2). The diagram was generated by BioEdit, version 7.0.1 (Informer Technology Inc., Los Angeles, CA).

**Figure 3:**
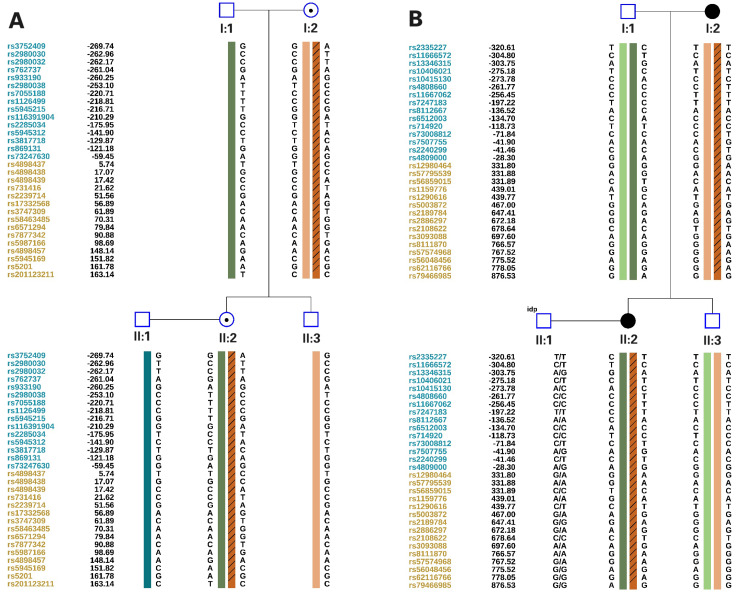
Single nucleotide polymorphism (SNP) linkage analysis of the family A: SNP linkage analysis of ABCD1; B: SNP linkage analysis of NOTCH3 The diagram was generated by ChromGo, version 1.9 (Yikon Genomics, Suzhou, China)

Before ovarian stimulation, with informed consent, 4 mL of peripheral blood was collected from family members I:1, I:2, II:1, II:2, and II:3, and genomic DNA was extracted using the EX-DNA whole blood genomic DNA extraction kit 3.0 (T146, Tianlong, China). Then the gDNA was hybridized with ASA chips (Infinium Asian Screening Array-24 v1.0 Kit, Illumina, San Diego, CA), and the arrays were scanned using an iScan system (SY-101-1001, Illumina). ChromGo software (Yikon Genomics, Suzhou, China) was used to analyze the scanned data, and the single nucleotide polymorphisms (SNPs) were genotyped. Haplotypes associated with disease-causing mutations were constructed based on informative SNPs within the 2 Mb region flanking ABCD1 and NOTCH3 (Figure 3), and a foundation was established for subsequent embryonic SNP linkage analysis.

**Figure 4:**
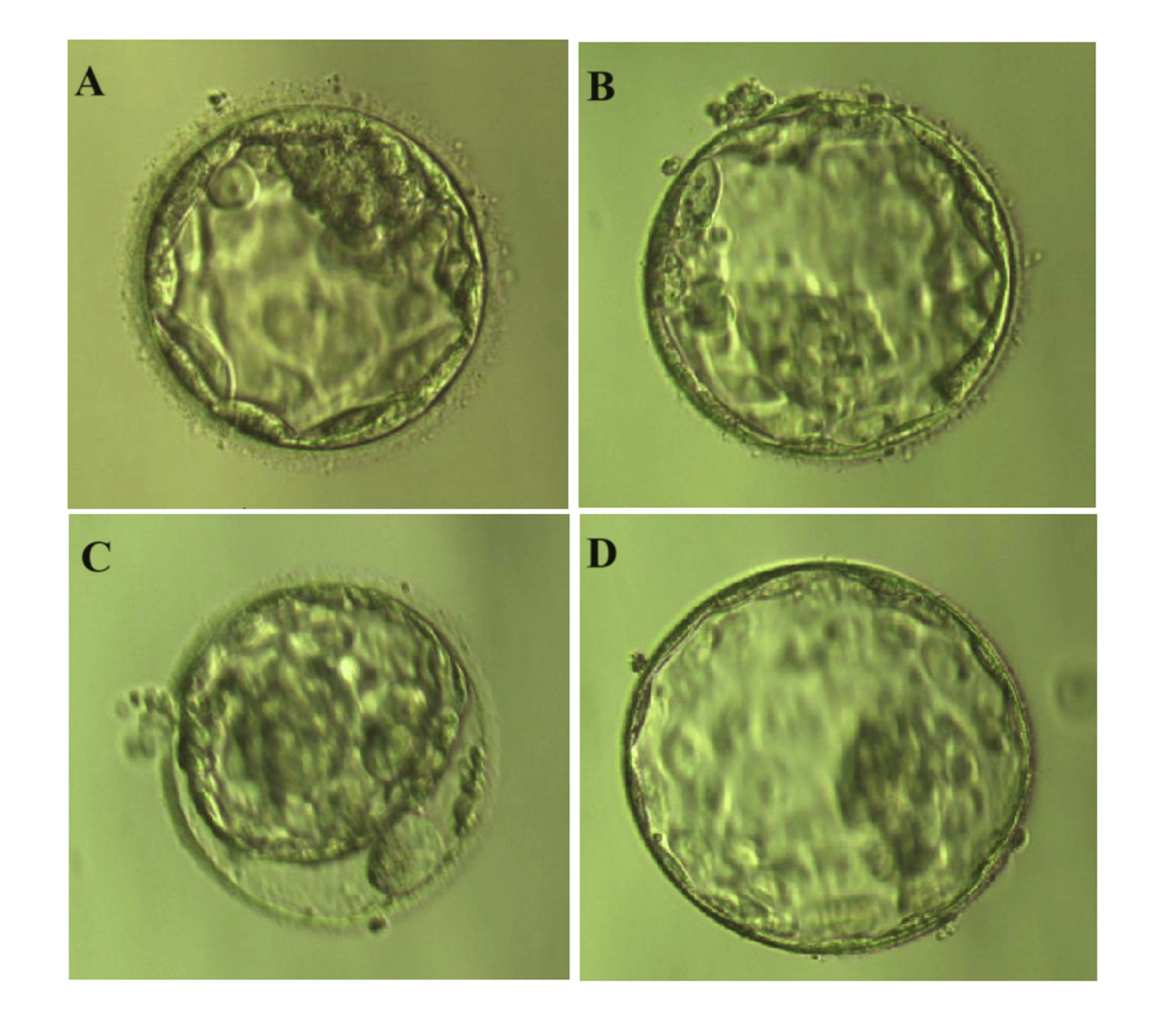
An image of the biopsied blastocyst under a stereoscope before the biopsy A: blastocyst E1; B: blastocyst E2; C: blastocyst E3; D: blastocyst E4. Blastocysts were observed under an inverted microscope (Ti-u, Nikon Instruments Inc., Melville, NY) and photographed using RI software (Research Instruments Ltd, Cornwall, UK).

**Figure 5:**
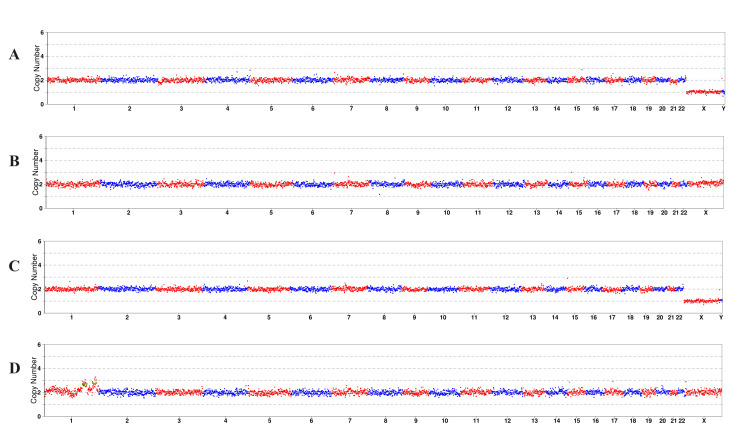
Whole genome copy number variations (CNV) schematic diagram of the biopsy blastocysts A: CNV schematic diagram of blastocyst E1; B: CNV schematic diagram of blastocyst E2; C: CNV schematic diagram of blastocyst E3; and D: CNV schematic diagram of blastocyst E4. The diagram was generated by ChromGo, version 1.9 (Yikon Genomics, Suzhou, China).

**Figure 6:**
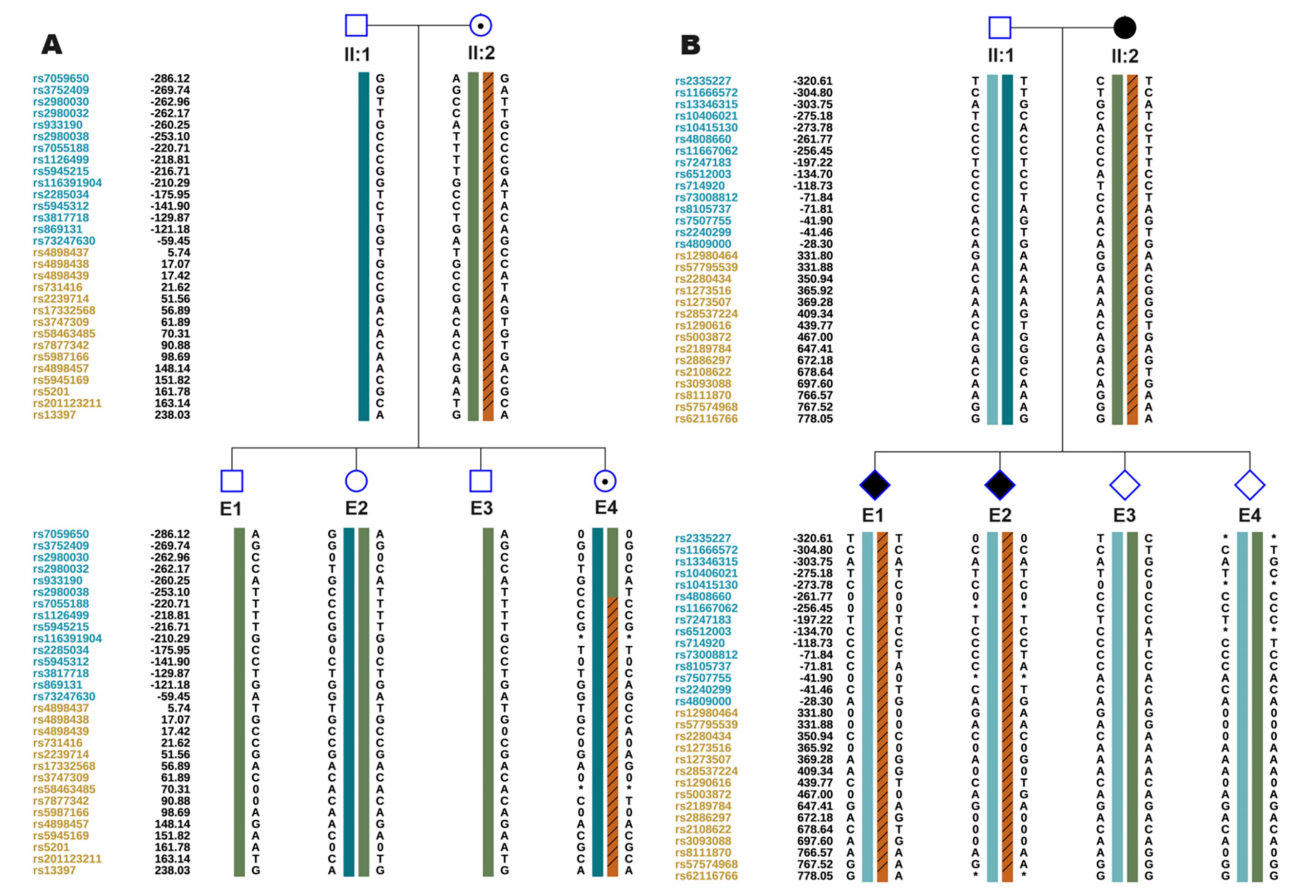
Single nucleotide polymorphism (SNP) linkage analysis of the biopsy blastocyst A: SNP linkage analysis of ABCD1; B: SNP linkage analysis of NOTCH3. The diagram was generated by ChromGo, version 1.9 (Yikon Genomics, Suzhou, China).

**Figure 7:**
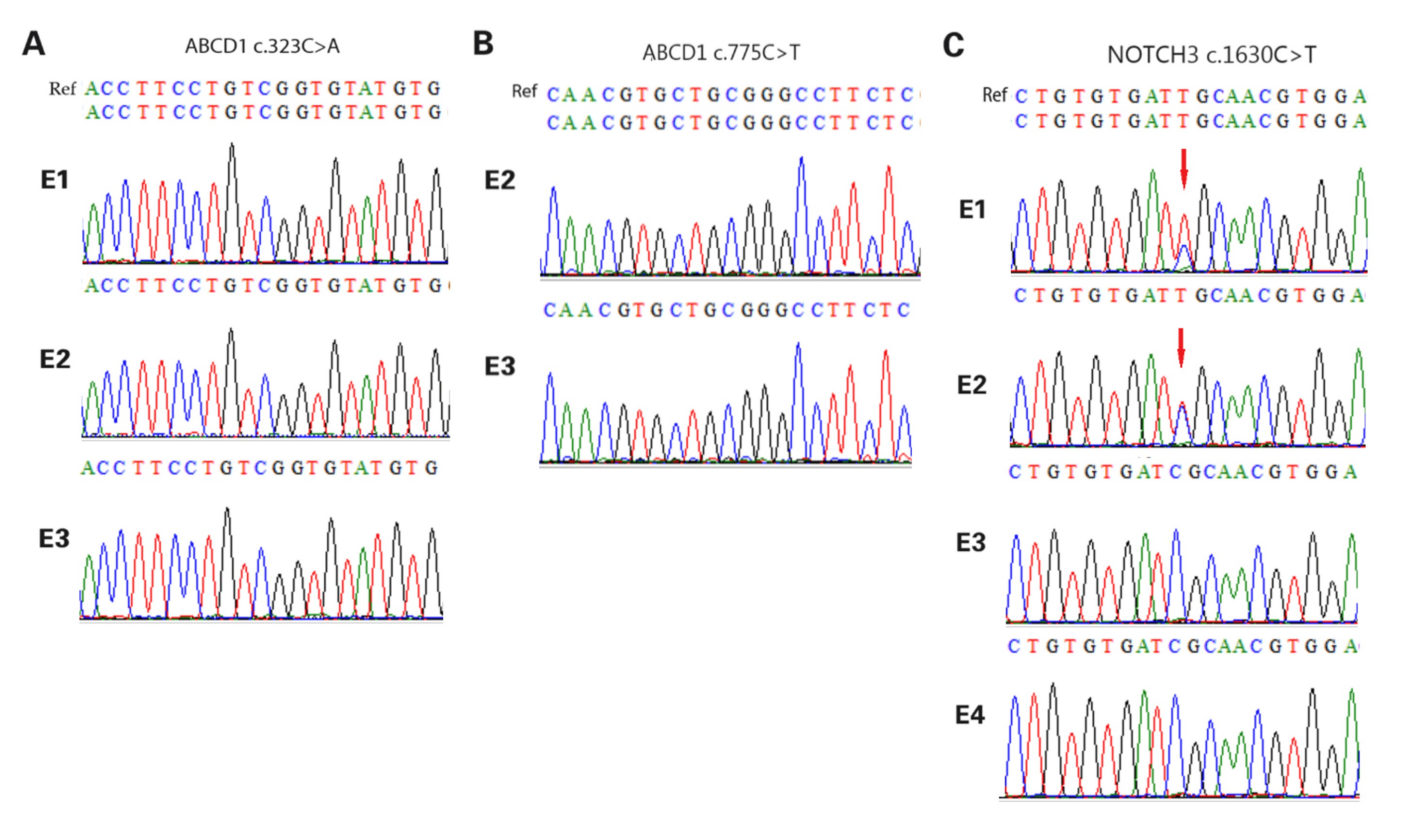
Sanger sequencing of pathogenic gene amplification products of biopsy blastocyst A: ABCD1 c.323C>A; B, ABCD1 c.775C>T; C, NOTCH3 c.1630C>T. The mutated positions are indicated by red arrows. The diagram was generated by BioEdit, version 7.0.1 (Informer Technology Inc., Los Angeles, CA).

We used an antagonist regimen to induce ovulation. The retrieved nine metaphase II (MII) oocytes were fertilized by intracytoplasmic sperm microinjection (ICSI) and cultured for five to six days in the in vitro fertilization (IVF) laboratory for blastocyst development. Five to 10 trophectoderm cells were removed from four blastocysts, referred to as E1, E2, E3, and E4 (Figure 4). These blastocysts were scored 4BC as per Garnder's criteria [8]. Whole genome amplification (WGA) was performed using a universal sample processing kit (ChromSwiftTM, XK-028; Yikon Genomics), according to the manufacturer’s instructions. The WGA products were purified using magnetic beads and fragmented using enzymes for library construction. The Illumina NextSeq 550 system (Illumina, San Diego, CA) was used for sequencing. To determine the embryonic genotypes, SNP haplotype analysis of the WGA-purified products and first-generation sequencing of the mutated loci amplification products was performed. Genetic testing revealed that E4 was an aneuploid blastocyst, whereas the other three were euploid blastocysts (Figure 5). Blastocysts E1 and E2 had the NOTCH3 heterozygous mutations, and E4 had the ABCD1 heterozygous mutations. E3 was the only euploid blastocyst that did not exhibit maternal genetic variations (Figures 6-7, Table 1). The E3 blastocyst was implanted into the mother’s uterus, and clinical pregnancy was achieved successfully. The prenatal amniocentesis results performed during the second trimester were consistent with the PGT results. The mother has recently given birth to a healthy male baby at full term.

**Table 1:** Genetic testing results of the biopsied blastocysts “/” indicates no mutation. The WGA products of E1 and E4 have poor coverage within the ABCD1 gene region, resulting in amplification failure and abnormal detection at mutational sites of ABCD1. CNV: copy number variations; SNP: single nucleotide polymorphism; WGA: whole genome amplification

No.	Days	Grade	CNV-seq	SNP linkage	Detection of mutational site		
					ABCD1, c.323C>A	ABCD1, c.775C>T	NOTCH3, c.1630C>T
E1	D5	4BC	Euploid	Not carrying maternal ABCD1 gene mutation, carrying maternal NOTCH3 gene mutation	/	Abnormal detection	Heterozygous mutation
E2	D5	4BC	Euploid	Not carrying maternal ABCD1 gene mutation, carrying maternal NOTCH3 gene mutation	/	/	Heterozygous mutation
E3	D6	4BC	Euploid	Not carrying maternal ABCD1 gene mutation and NOTCH3 gene mutation	/	/	/
E4	D6	4BC	dup(1)(q25.3q32.1)(~20.80Mb),dup(1)(q42.11q43)(~17.60Mb)	Carrying maternal ABCD1 gene mutation, not carrying maternal NOTCH3 gene mutation	Abnormal detection	Abnormal detection	/

## Discussion

X-linked adrenoleukodystrophy (X-ALD) is an X-linked recessive hereditary disease caused by mutations in ABCD1, with a prevalence of 1:17000 [2]. Approximately 65%-80% of female carriers develop AMN symptoms at older ages [2]. According to the http://adrenoleukodystrophy.info database, to date, more than 4,200 ABCD1 variations have been identified, including over 3,600 (primarily missense mutations) pathogenic and potentially pathogenic mutations. Approximately 95% of the affected individuals inherit the ABCD1 pathogenic variant from one of their parents, and an estimated 4.1% of the index cases are caused by a de novo pathogenic variant [9]. There is no definite correlation between the X-ALD genotype and phenotype [1]. The same mutation may lead to the development of varying clinical manifestations, onset times, and disease severity. Although extensive research has been conducted on X-ALD in various disease models (mice, flies, nematode, and zebrafish models), the pathological and physiological mechanisms have not been elucidated [1,2]. To date, animal models have presented partial AMN phenotypes, whereas some important pathological phenotypes of cALD, such as demyelination, blood-brain barrier disruption, and the presence of macrophages and lymphocytes in the perivascular space of the central nervous system, have not been described [1,2]. Curiel et al. [10] reported the case of a chimpanzee in captivity who presented with classic clinical and MRI-confirmed cerebral X-ALD, with ultimate confirmation of the diagnosis via VLCFA analysis and ABCD1 sequencing. This suggests that chimpanzees could be a suitable model for cALD.

Currently, X-ALD therapies include bone marrow transplantation, allogeneic hematopoietic stem cell transplantation, lentiviral hematopoietic stem cell gene therapy, and dietary therapy to reduce VLFCA levels [1]. These treatments offer limited therapeutic effects, and each method is associated with drawbacks. More studies on X-ALD are needed to elucidate its associated pathological and physiological mechanisms and develop novel, safe, and effective therapeutic strategies.

A progressive disease, CADASIL, is associated with vascular abnormalities affecting arterial vascular smooth muscle cells (VSMCs), is an autosomal dominantly inherited disease caused by a NOTCH3 mutation. Depositions of granular osmiophilic material (GOM), which includes NOTCH3 ECD and other components, occur in the VSMC basement membrane, and there is degeneration and loss of VSMCs through apoptosis or altered proliferation [6]. The pathological mechanism of CADASIL remains elusive, and there is no consensus on how NOTCH3 mutations lead to the occurrence of CADASIL. The debate between different viewpoints mainly focuses on whether defects in NOTCH3 result in changes in the NOTCH3 signal and whether changes in the NOTCH3 signal lead to CADASIL [4,6,11].

The incidence of CADASIL is estimated at two to five in 100,000 [11], varying between populations. More than 90% of pathogenic variants of NOTCH3 associated with CADASIL occur within exons 2-23, leading to a gain or loss of cysteine residues within the EGF repeat regions of NOTCH3, which results in an abnormal protein conformation of NOTCH3 [4-5,11]. Pathogenic variations in EGFr domains 1-6 appear to be completely exomic, usually associated with typical CADASIL phenotypes, whereas pathogenic variations in epidermal growth factor receptor (EGFR) domains 7-34 display a higher population frequency, and the phenotype may be mild or even non-dominant [12-13]. In addition, the relationship between CADASIL genotype and phenotype indicates that the spectrum of NOTCH3 mutations varies among different ethnic races, and distinct NOTCH3 mutational profiles and genetic backgrounds may give rise to different clinical phenotypes [14]. In summary, the findings indicate that different mutational spectra are responsible for the phenotypic variations observed among different ethnic groups. Currently, in clinical practice, interventions only target specific symptoms, and an effective therapy for CADASIL remains elusive.

Given the current research on the pathological mechanisms and therapies for X-ALD and CADASIL, prenatal diagnosis and preimplantation genetic testing are the best methods for inhibiting disease transmission. Lledó et al. [15] reported a case of a female who received preimplantation genetic testing. In this study, to assess the status of embryos, multiple displacement amplification (MDA) was used for WGA, and two extragenic polymorphic markers (DXS1073 and DXS9901) that flank ABCD1 as well as X22, an X/Y chromosome marker, were used for linkage analysis. Unfortunately, the patient was not pregnant following the transplantation of three unaffected female embryos. In another case reported by Son Trinh [16], PGT-M was conducted in a Vietnamese family; the segment spanning the detected mutation was sequenced by Sanger sequencing, and embryos that did not possess mutated alleles were screened for chromosomal abnormalities using next-generation sequencing (NGS). Based on this pattern, the carrying status and gender of the embryos were analyzed. The couple successfully conceived and delivered a healthy female infant at full term. There are few reports on PGT for NOTCH3 mutation carriers, which may be due to the late CADASIL onset. Konialis et al. [17] described an affected father who carried a NOTCH3 disease-causing mutation. Short tandem repeat (STR) markers and an intragenic SNP, coupled with mutation identification, were used for embryo analysis. Three unaffected embryos were unambiguously identified among the eight biopsy embryos. Blastocyst transfer resulted in a singleton pregnancy, and a subsequent prenatal diagnosis confirmed that the fetus was disease-free.

In our study, c.323C>A (p.Ser108 *), c.775C>T (p.Arg259Trp) in ABCD1, and c.1630C>T (p.Arg544Cys) in NOTCH3 were found in the family affected by white matter disease. ABCD1 c.323C>A is a rare nonsense mutation that is not included in the gnomAD East Asian general population database. The mutation may result in amino acid loss of greater than 10%, leading to the occurrence of nonsense-mediated mRNA decay (NMD). This may be a non-functional mutation, as one of the pathogenic mechanisms of ABCD1 is loss of function. Several studies have reported this variant in children with adrenal leukodystrophy [18-20]. According to the guidelines of the American College of Medical Genetics and Genomics (ACMG), this variant is classified as pathogenic. ABCD1 c.775C>T (p.Arg259Trp) is a rare variant with a frequency of zero in the East Asian GnomAD general population database. A variety of bioinformatics software packages predict that this variant will have detrimental effects on genes or gene products. According to the ACMG guidelines, it is a "variant of uncertain significance". NOTCH3 c.1630C>T is a missense mutation with a high frequency of 0.003967 in the gnomAD East Asian general population database. This variant was found in over 155 patients with CADASIL, some of whom were homozygous, and subgroup analysis showed that this mutation is associated with a lower frequency of anterior temporal involvement, later onset age, and a higher frequency of cognitive impairment. Hence, this variant has been hypothesized to exhibit a founder effect in Asians and is the most common variant associated with CADASIL in East Asian populations. In addition, this variant introduces a cysteine residue between the EGFr-13 and EGFr-14 domains, which is different from the cysteine residue involved in other mutations, leading to a milder conformational change in NOTCH3, resulting in a milder clinical phenotype. According to the ACMG guidelines, it is a possible pathogenic variant [13,21-23].

The clinical manifestations of cALD and CADASIL partially overlap, such as white matter damage, gait instability, and memory loss, which are presented by the proband in this case. Although doctors are more inclined to diagnose the proband as cALD, the genetic risks associated with variants in NOTCH3 cannot be ignored. After genetic counseling, the patient chose to block the transmission of pathogenic variants in both ABCD1 and NOTCH3 to their offspring simultaneously.

## Conclusions

To the best of our knowledge, this is the first report of the presence of both ABCD1 and NOTCH3 mutations in a family with cerebral white matter disease. As the partially overlapping clinical manifestations of X-ALD and CADASIL, adequate genetic counseling and risk notification are particularly important in the diagnosis and treatment process. This allows patients to choose the most beneficial fertility method based on understanding all genetic risks. Our research confirms the feasibility of PGT in blocking intergenerational transmission of the ABCD1 and NOTCH3 mutations, which have important clinical applications in the prevention and control of birth defects.
